# The Effect of Deep Brain Stimulation Therapy on Fear-Related Capture of Attention in Parkinson’s Disease and Essential Tremor: A Comparison to Healthy Individuals

**DOI:** 10.4172/2329-6895.1000377

**Published:** 2018-02-27

**Authors:** Corrie R Camalier, Maureen McHugo, David H Zald, Joseph S Neimat

**Affiliations:** 1 Department of Neurosurgery, Vanderbilt University Medical Center, Nashville, TN, USA; 2 Laboratory of Neuropsychology, National Institute of Mental Health (NIMH), Bethesda, MD, USA; 3 Department of Psychiatry and Behavioral Sciences, Vanderbilt University Medical Center, Nashville, TN, USA; 4 Departments of Psychology, Psychiatry and Behavioral Sciences, Vanderbilt University Nashville, TN, USA

**Keywords:** Parkinson’s disease, Essential tremor, Deep brain stimulation, Emotional blink, Attention, DBS, STN, VIM

## Abstract

In addition to motor symptoms, Parkinson’s disease (PD) involves significant non-motor sequelae, including disruptions in cognitive and emotional processing. Fear recognition appears to be affected both by the course of the disease and by a common interventional therapy, deep brain stimulation of the subthalamic nucleus (STN-DBS). Here, we examined if these effects extend to other aspects of emotional processing, such as attentional capture by negative emotional stimuli. Performance on an emotional attentional blink (EAB) paradigm, a common paradigm used to study emotional capture of attention, was examined in a cohort of individuals with PD, both on and off STN-DBS therapy (n=20). To contrast effects of healthy aging and other movement disorder and DBS targets, we also examined performance in a healthy elderly (n=20) and young (n=18) sample on the same task, and a sample diagnosed with Essential Tremor (ET) undergoing therapeutic deep brain stimulation of the ventral-intermediate nucleus (VIM-DBS, n=18). All four groups showed a robust attentional capture of emotional stimuli, irrespective of aging processes, movement disorder diagnosis, or stimulation. PD patients on average had overall worse performance, but this decrement in performance was not related to the emotional capture of attention. PD patients exhibited a robust EAB, indicating that the ability of emotion to direct attention remains intact in PD. Congruent with other recent data, these findings suggest that fear recognition deficits in PD may instead reflect a highly specific problem in recognition, rather than a general deficit in emotional processing of fearful stimuli.

## Introduction

Parkinson’s disease (PD) is a neurodegenerative movement disorder that also has significant and increasingly appreciated non-motor symptoms. For example, patients with PD exhibit deficits in the recognition of emotion, particularly in the recognition of fear and disgust [1–14]. The source and extent of these recognition deficits is unclear, as some early components of emotion processing appear spared [15–17]. It is also unclear to what degree a common neurosurgical therapy, deep brain stimulation of the subthalamic nucleus (STN-DBS), affects these emotional deficits. Some studies report impaired fear recognition to faces following STN-DBS [10,18–20], which suggests that emotion recognition is affected by stimulation of the affected motor structures in PD (possibly via degradation of the limbic loop of the basal ganglia [21].

To bring new light to the understanding of the nature of these deficits, we turn to emotion’s ability to route attentional resources. In healthy individuals, highly emotional stimuli such as those conveying threat, “capture” attention. This capture of attention is commonly studied using the emotional attentional blink (EAB) paradigm [22]. In this, the presentation of a task-irrelevant, strongly emotional distractor image transiently impairs the ability to detect a target presented later. Given the evidence for fear-related emotion recognition deficits in PD, it seems reasonable to ask if emotional capture of attention is impaired in PD, and if therapeutic STN-DBS. affect it the prediction is if key processes involved in emotion recognition and the EAB are shared, then one would expect a reduced EAB in PD relative to controls. By contrast, if aspects of emotion recognition and attentional capture rely on different processes, the EAB may be intact relative to controls. Additionally, if STN-DBS were shown to affect the magnitude of the EAB, then it would suggest that emotion’s ability to capture attention and emotion recognition share common processing substrates. An intriguing alternative possibility to the hypothesis that emotion deficits are from degradation of the limbic loop in PD [21] is that the emotion deficits are instead tied to deficits in movement processing. Emotion is a powerful modulator of behavior, and emotional experience is often tied to the modulation of motor system function. In humans, highly emotional images, both appetitive and aversive, increase motor system excitability [23], and deficits in emotional processing in PD have been taken as evidence for the tight coupling of motor and emotional processing. Thus, a complementary aim of this study was to test whether motor disruptions due to other movement disorders and DBS stimulation of other motor regions will affect the EAB. Essential Tremor (ET) is a movement disorder characterized by tremor of the arms, hands, and other body parts during intentional movement. Supporting the suggestion that emotion and motor structures may be linked, ET patients may also exhibit subtle emotion impairments, such as mood dysregulation [24,25]. DBS of the ventral-intermediate nucleus (VIM-DBS), a motor nucleus of the thalamus, is used to improve symptoms of ET, and there are reports of anxiety/fear affected by VIM-DB [26].

Thus, we compared the EAB across four groups, leading to several predictions. First if the emotional capture of attention is preserved in the elderly, one should expect to see similar magnitude of emotional capture of attention in healthy young and aged. This establishes an important validity of the task in elderly groups such as PD and ET. Second, if the emotional capture of attention is dependent on similar processes as fear recognition, then we expect a reduced emotional capture of attention in PD than in healthy aged, and for it to be affected by therapeutic STN-DBS. If the emotion disruptions are due instead to general motor disruptions, then we should expect to see a reduced EAB in essential tremor, additionally affected by VIM-DBS. In contrast, if EAB deficits are specific to basal ganglia degradation, then we should expect to see no such disruption of attentional capture in ET.

## Materials and Methods

### Subjects

Four groups of subjects participated in this study (Table 1): 1) Healthy young controls (HYC, n=18), 2) Healthy elderly controls (HEC; n=20), 3) Parkinson’s disease patients undergoing therapeutic bilateral deep brain stimulation of the subthalamic nucleus (PD STN-DBS; n=20), 4) Essential tremor patients undergoing bilateral deep brain stimulation of the motor thalamus ventral-intermediate nucleus (ET VIM-DBS; n=18). Parkinson’s and Essential tremor patients were recruited from the Vanderbilt University movement disorders clinic, healthy elderly was recruited from the local community, and healthy young were recruited from the Vanderbilt student body. Subjects had no history of neurological deficits (e.g. stroke) or major psychiatric conditions (e.g. bipolar disorder). The elderly groups were screened for dementia or other broad cognitive decline by a comparison of the current (Wechsler Abbreviated Scale of Intelligence WASI: [27], combined vocabulary and matrix reasoning subtests) to estimated premorbid IQ (Wechsler Test of Adult Reading, WTAR: [28] – if the difference was greater than 25 points, the subject was excluded from the study as this would suggest a substantial decline from premorbid IQ. All groups matched for education (ANOVA, education by group: F(3)=2.1, p=0.11). The elderly groups matched for age (ANOVA, age by group: F(2)=1.2, p=0.32). Groups were similar in IQ, except that the HEC IQ was modestly but significantly higher than both the PD and ET patient groups (ANOVA, IQ by group: F(3)=4.9, p=0.003; Tukey post-hoc comparisons, HEC *vs.* PD, p=0.01, HEC *vs.* ET, p=0.01, all other p>0.05). Each participant gave written informed consent, and all procedures were in accordance with and approved by the Vanderbilt Institutional Review Board (IRB #111730, 171210).

### PD and ET patient characteristics and deep brain stimulation settings

ET and PD groups had bilateral quadripolar DBS electrodes implanted into either the STN (for PD) or VIM (for ET), according to surgical procedures published previously [29]. All patients were tested with stimulation settings used to achieve optimal clinical benefit of motor symptoms, determined by their Vanderbilt movement disorders neurologist (location and settings, Table 2). For the PD group, time since DBS implantation surgery was 25.5 months (standard deviation (S.D.)=24.7), years since diagnosis was 10.0 (6.4). They were tested on Levodopa medications, average daily dose 940 mg (672), and conversion after [30]. Patients were Hoehn and Yahr stage 3-4. For the ET group, time since DBS implantation surgery was 35.8 months (46.2). Due to the gradual progression of essential tremor, time since diagnosis was not available. At the time we did this experiment, DBS patients were not routinely screened for postoperative motor “ON” efficacy scores at our center, though all patients reported proper control of motor symptoms. Consistent with this, AC-PC coordinates of center of active DBS contact (requiring a post-operative CT merged with a preoperative structural MRI) was available for most of the patients (19/20 PD, 16/18 ET, Table 2). Note that lead location is consistent across patients.

### Task and procedure

**Figure 1:**
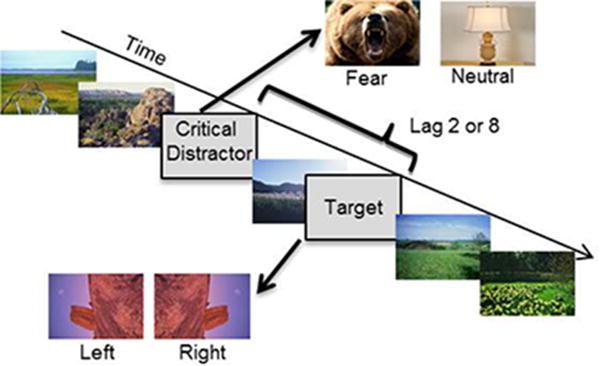
Emotional attentional blink task design. Subjects watched a rapid serial visual presentation (RSVP) stream of upright images for a target rotated image. Either 2 or 8 images before the target image, a distractor images were presented that was either neutral (lamp) or fear-inducing (bear). At the end of the RSVP stream the reported the direction of the rotated target.

Subjects performed an EAB task in which they were instructed to monitor a rapid serial visual presentation (RSVP) stream of upright images for a rotated image (Figure 1). Targets were 120 rotated landscape/architectural photos; half were rotated 90 degrees to the left and half were rotated 90 degrees to the right. Within the RSVP stream there were two types of non-target images: standard images −256 upright landscape/architectural photos, and critical distractors −40 images consisting of 2 categories (20 fear, 20 neutral). Fear pictures included animals bearing teeth in a threatening manner, humans brandishing weapons, and explosions. Neutral pictures included images of tables, lamps, and plants. Critical distractor images were taken from the International Affective Picture System [31], supplemented with images from publicly available online sources. Valence and arousal ratings were not obtained from individual subjects in this experiment due to time limitations, but these images have been used in previous EAB paradigms within the lab and generally induce a strong EAB.

Each session contained 120 trials; in half of these trials the critical distractor conveyed fear/threat and the other half were neutral. On each trial, a critical distractor appeared in the 4th, 6th, or 8th position in the RSVP stream). A rotated target appeared 200 or 800 ms (lag 2 or 8) following the critical distractor. The critical distractor and target rotation were fully counterbalanced within a session. At the end of the RSVP stream, subjects were asked to indicate by a no speeded key press or verbal response whether they detected a target rotated to the left, right or if a target was absent. Before the experimental session began, subjects completed at least 10 practice trials in which no critical distractor was presented. The task was programmed in E-Prime 1.2 (Psychology Software Tools, Pittsburgh, PA). For the STN- and VIM-DBS groups, each participant had two sessions within the same day: bilateral stimulation ON vs, OFF. Stimulation order was counterbalanced within each group, and at least 15 minutes could elapse after change of stimulation settings [32]. Images in the RSVP stream were presented every 100 ms and remained on screen for that time. However, during piloting, the initial cohort of PD STN-DBS patients (n=6) performed at chance (~50% accuracy) in the neutral control lag 8 condition (and all other conditions), indicating that the presentation duration was too fast for the patients to accurately see any of the targets. Consistent with adjustments made in other studies with patient populations [33], for the PD group only we increased presentation duration to 120 ms. This minor extension is necessary since the measure of interest was whether attention is differentially captured following an emotional stimulus and intact performance on the neutral condition at lag 8 was an important prerequisite. A separate 20 PD patients were recruited and run on this improved version.

### The EAB measure and analysis

The EAB is a substantial decrement in detection accuracy when the rotated target is presented quickly after a threatening image (lag 2) relative to when the target is presented later in the stream (lag 8) or following a neutral image at any lag. To measure it, proportion of correctly detected target rotation is calculated for each emotion (fear/neutral) and lag (2/8) condition. To determine if the EAB is present in a given group, the comparison of interest is an emotion × lag interaction. As a secondary measure for comparing performance between groups, we calculated “blink amount” defined as the difference in accuracy between the lag 2 neutral and fear condition, also called “disengagement efficiency index [34]. This measure provides an index of emotion induced capture of attention at a single point in time and does not depend on how performance recovers over time. For all analyses, we performed appropriate analyses of variance analyses (ANOVAs) with posthoc Tukey tests to examine group differences, if any. Stimulation order (ON/OFF DBS) was fully counterbalanced within and across groups, but as a control, we re-ran analyses with stimulation order as an additional factor and no effects changed. As a further additional control to examine habituation effects, we examined the emotion accuracy for the PD and VIM groups (who both ran two sessions), split by session half (first half of session *vs.* second half of session). Session half or any interaction with it was not significant; yielding further evidence that habituation was not a factor in the experiment. Statistical analysis was performed with SPSS (Armonk, NY), and criteria for significance was set such that α=0.05.

## Results

### Validation of the EAB in the elderly

**Figure 2:**
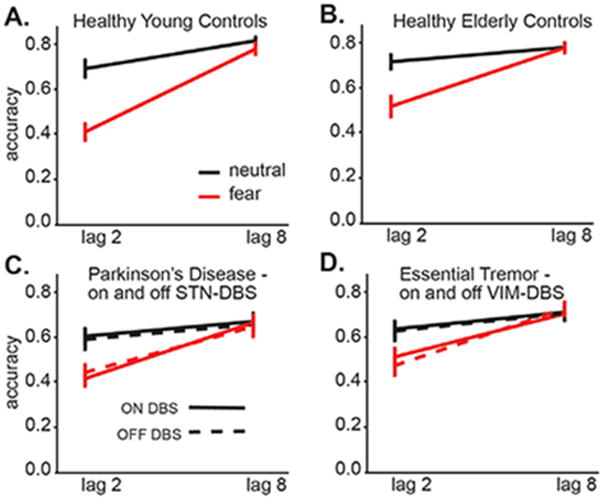
Performance (accuracy, expressed as proportion correct) for lags and emotion conditions for **A:** healthy young, **B:** healthy elderly, **C:** the Parkinson’s disease ON and OFF bilateral STN-DBS and **D:** Essential Tremor ON and OFF VIM-DBS. Note that all groups show substantial decrement in fear lag 2 relative to all other conditions.

To first establish the validity of the EAB paradigm in the healthy elderly, we compared performance between matched cohorts of healthy aged (HEC) and healthy young (HYC). (Figures 2A and 2B) show target detection performance for these groups and note that both groups exhibit a robust EAB: a substantial decrement in performance when the rotated target is presented quickly after a threatening image (lag 2) relative to when the target is presented later in the stream (lag 8) or following a neutral image at any lag. This similarity in performance validates this paradigm in the elderly. These effects were confirmed by a 2 × 2 × 2 (emotion × lag × group) mixed within/between subjects ANOVA (emotion: F(1,36)=96.5, p<0.01, lag: F(1,36)=115.0, p <0.01, group: F(1,36)=0.63, p>0.05; no interaction terms reached significance except emotion × lag, F(1,36)=42.7, p<0.01), indicating a fear-based emotional blink of attention.

### The EAB is unaffected by movement disorder diagnosis and DBS therapy

Figure 2C shows performance in the PD group both ON and OFF STN-DBS stimulation. An attentional blink was seen following the threat images; however, STN-DBS stimulation did not affect performance in any condition. These effects were confirmed by a 2 × 2 × 2 × 2 (emotion × lag × stimulation × stimulation order) mixed within/between subjects ANOVA (emotion: F(1,18)=20.7, p<0.01, lag: F(1,18)=44.2, p<0.01, stimulation: F(1,18)=0.04, p>0.05, order F(1,18)=1.3, p>0.05, no interaction terms reached significance except emotion × lag, F(1,18)=36.4, p<0.01), indicating a fear-based emotional blink of attention. Figure 2D shows performance in the ET group both ON and OFF VIM-DBS stimulation. Like the PD group, they also showed a robust EAB that is unaffected by DBS therapy. These effects were confirmed by a 2 × 2 × 2 × 2 (emotion × lag × stimulation × stimulation order) mixed within/between subjects ANOVA (emotion: F(1,16)=25.4, p<0.01, lag: F(1,16)=33.8, p<0.01, stimulation: F(1,16)=0.56, p>0.05, order F(1,16)=0.91, p>0.05, no interaction terms reach significance except emotion × lag, F(1,16)=31.0, p<0.01).

**Figure 3:**
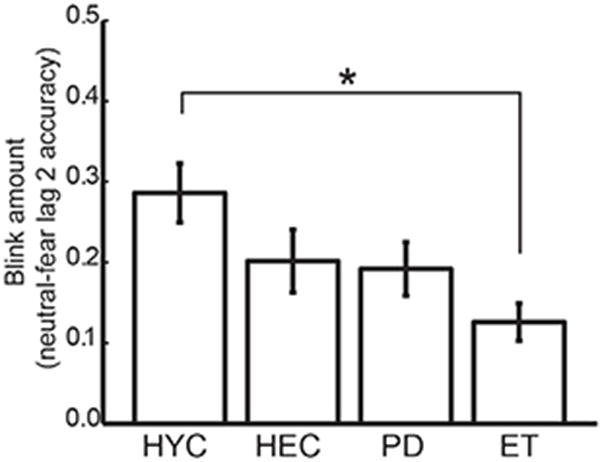
Blink amount (visualized as difference in accuracy between lag 2 neutral vs lag 2 emotion) for each group: healthy young (HYC), healthy elderly (HEC), the Parkinson’s disease ON bilateral STN-DBS (PD), and Essential Tremor ON VIM-DBS (ET). Error bars denote standard error of the mean. Post hoc comparison indicates the only group comparison that shows a significantly different blink amount is VIM and HYC, denoted by a star.

Overall, each group exhibited patterns of performance consistent with an emotional attentional blink, suggesting that it can be induced irrespective of movement disorder diagnosis, age, or DBS therapeutic state. It is possible that the blink amount, the difference in accuracy between the lag 2 neutral and fear condition, would differ between groups (see Methods). For example, if PD patients are less affected by emotional stimuli (e.g. due to a deficit in recognizing emotion) they would be less distractible, and blink amount would be less than other groups. Blink amount was compared between the HEC, HYC, and the presumably optimal state of the PD and ET groups, both in the DBS-ON condition. This is visualized in Figure 3 as the mean of each subject’s differences in accuracy between lag 2 neutral *vs.* lag 2 emotions (the “blink amount”) for each sample. Overall blink amount significantly differed between groups, an effect driven by the difference between HYC and ET, but there was not a significant interaction of group and emotion, indicating that performance in the emotional condition did not differ between groups relative to the control condition (2 × 4 (emotion × group) mixed within/between subject ANOVA on blink amount (effect of emotion F(1,72)=85.1, p<0.01, group F(3,72)=3.4, p=0.02, no interaction, Tukey posthoc tests n.s. except for HYC *vs.* ET p=0.02). Thus, groups did not appear to differ in the amount of emotional attentional blink that these images induced, despite differences in movement disorder diagnosis, therapeutic state, and age.

## Discussion

We examined whether individuals with PD show reduced threat-based emotional attentional blink consistent with reports of reduced fear recognition. Further, we examined the effect of therapeutic STN-DBS on attentional blink magnitude to understand the effects of neuroanatomically precise interventions on this measure. We also compared the existence and magnitude of the EAB in the healthy elderly, healthy young, and individuals with ET on and off therapeutic VIM-DBS. Contrary to expectations, all four groups showed an emotional blink, irrespective of aging processes or movement disorder diagnosis. PD patients, on average, had overall poorer performance, even with a slightly slower version of the task. This decrement in performance was neither emotion nor lag specific and thus was unrelated to the stimulus driven capture of attention but was instead probably due to general cognitive slowing in this population [35]. These findings help constrain the range of features in affective processing that are altered in PD. Rather than a broad deficit in affective processing, PD may impact recognition of certain emotions in faces, voices and other mediums, but not the ability of emotional stimuli to capture attention. In considering this difference, it is useful to consider the involuntary, stimulus driven nature of the emotional attentional blink. The task does not require speeded movements (including eye movements), and EAB existence does not depend on goal directed attentional mechanisms. As such, our data are consistent with studies reporting normal early responses to emotional images in PD, such as the pupillary response and the early posterior negativity [12,15].

In addition to no differences in magnitude across groups, the magnitude of the EAB was unaffected by therapeutic DBS. Several reports suggest STN-DBS can affect emotional processing, such as emotional face recognition [10,18,19]. This dissociation between deficits in explicit fear recognition shown previously and intact performance in more implicit tasks such as the EAB shown in the present study suggest that the course of the disease and therapeutic condition may differentially affect some emotional processing paths. Indeed, while STN-DBS therapy appears to have effects on some aspects of executive functions broadly defined, which include some measures of attention [36–39], there appear to be no DBS effects on an emotional image’s power to siphon attentional resources, consistent with the automatic stimulus-driven nature of this phenomena. In ET patients, the EAB was also unaffected by therapeutic VIM-DBS. This group is an ideal population with which to compare PD performance, as they are both elderly movement disorder groups undergoing therapeutic stimulation of motor-related structures with similar neurosurgical processes used for implantation. The finding that neither STN- nor VIM-DBS affect the EAB suggests that while some aspects of emotion may be tightly linked to the motor system, modulating the motor system per se does not have an obligatory effect on the allocation of attention resources to threatening images; nor does therapeutic deep brain stimulation of the STN or VIM, or the neurosurgical process per se, produce untoward effects on these processes.

One important caveat to this study is that stimuli typically used for the EAB (threatening images of humans and animals), are different from those used for emotion recognition (often, but not exclusively, faces). In contrast to the results with emotional images [12], the EPN measure of early processing, has been reported to be abnormal in response to faces in PD [16], which may suggest differences in the way that facial *vs.* other emotional stimuli are processed [40], again suggesting that the range of affective disturbance in PD may be restricted. Images used in this study were optimal to examine disease and stimulation effects on fear-based capture of attention, as faces are generally only weak emotional inductors of the EAB [41]. Nevertheless, the fact that we did not collect data regarding emotional faces limits our ability to determine what features more precisely allow the EAB to be preserved in PD patients. It would be an interesting extension to test recognition of emotional faces and the EAB in the same sample of patients to determine if EAB responses are truly dissociable from emotion recognition deficits. While our data make clear that EAB is generally intact in PD, evidence for dissociation would require examination of EABs in patients with demonstrable deficits in emotional recognition. In addition, it may be noted that the PD patients in this study were reasonably high functioning in that their mean current IQ was in the average range and we excluded cases where there was evidence of dementia after review of medical records and our own IQ testing. Thus, the results may not generalize to PD patients with severe cognitive deficits. However, given that the patients in the study had severe enough symptoms to warrant STN-DBS, the level of PD symptoms was clearly substantial and representative of the common expression of PD. Critically the preservation of the EAB suggests that to the extent that either PD or STN-DBS are related to cognitive deficits, they are not interfering with the expression of the EAB.

## Conclusion

In summary, this study shows that despite previous reports of deficits in fear recognition, PD patients still show a robust fear-based EAB. The inclusion of the EAB task to the growing literature examining emotional function in PD allows greater specificity in understanding the nature of emotional deficits, as it does not rely on nonemotionally processing components known to be affected by PD, such as eye movements. In addition, it suggests that stimulation of common neurosurgical targets for DBS, such as the VIM and the STN, do not affect measures of fear impacting attentional resources.

## Figures and Tables

**Table 1:** Demographic information (mean (standard deviation)) for young and elderly controls (HYC and HEC, respectively), and Parkinson’s (PD) and Essential tremor (ET) subjects.

Group	n	Current IQ	Yrs Education	Gender (# males)	Age (yrs)	Handedness (# right)
HYC	18	107.9 (5.5)	13.9 (1.0)	5	21.0 (4.9)	17
HEC	20	117.8 (11.3)	15.7 (2.1)	11	64.9 (8.1)	19
PD	20	105.8 (14.4)	14.7 (2.3)	14	60.8 (9.3)	18
ET	18	104.8 (13.5)	14.6 (2.7)	12	62.6 (9.3)	18

**Table 2:** DBS settings and AC-PC coordinates of center of active DBS contact (mean (standard deviation)) for all patients available (STN:19/20, VIM:16/18), listed separately by hemisphere.

Variables	Voltage, V	Pulse width, μs	Frequency, Hz	Lateral, mm	Posterior, mm	Superior, mm
STN – Left	2.3 (0.98)	70.5 (14.7)	126.5 (15.7)	11.6 (1.3)	2.1 (1.8)	−3.1 (2.2)
STN – Right	2.4 (0.80)	72.0 (15.1)	126.5 (15.7)	−11.2 (1.1)	1.3 (2.0)	−2.3(1.8)
VIM – Left	3.0 (1.20)	99.3 (27.9)	137.1 (16.9)	13.9 (1.4)	5.2 (2.8)	4.2 (3.0)
VIM – Right	2.5 (1.40)	90.0 (27.2)	140.0 (20.4)	−14.9 (2.0)	4.6 (2.8)	4.6 (3.4)
